# Starvation Ketoacidosis With Hypoglycemia in a Patient With Chronic Pancreatitis

**DOI:** 10.7759/cureus.12756

**Published:** 2021-01-17

**Authors:** Masakazu Kakurai, Hiroshi Ito, Nako Matsumoto, Nobutake Shimojo, Satoru Kawano

**Affiliations:** 1 Division of Hospital Medicine, University of Tsukuba Hospital, Tsukuba, Ibaraki, JPN; 2 Department of Endocrinology and Metabolism, University of Tsukuba Hospital, Tsukuba, Ibaraki, JPN

**Keywords:** starvation ketoacidosis, hypoglycemia, chronic pancreatitis

## Abstract

Chronic pancreatitis is a pancreatic inflammation that can result in endocrine pancreatic insufficiency. We present a case of starvation ketoacidosis in a 44-year-old Japanese man with chronic alcoholic pancreatitis. On admission, he exhibited hypoglycemia and severe acidosis. Intravenous glucose and vitamin B1 were administered in the emergency department, and nutritional management for presumed starvation ketoacidosis was begun. Because the patient did not have diabetes mellitus, his insulin secretion and insulin resistance were examined. A diagnosis of pancreatic diabetes caused by chronic pancreatitis was made based on decreased insulin secretion, normal insulin resistance, and negative anti-glutamic acid decarboxylase antibody. Intensive insulin therapy was initiated, and he was discharged 15 days after hospitalization. Although starvation rarely causes hypoglycemia and severe ketoacidosis, they can be induced by short-term fasting in patients with decreased pancreatic function.

## Introduction

In chronic pancreatitis, a multifactorial, fibroinflammatory syndrome, repeated episodes of pancreatic inflammation results in endocrine pancreatic insufficiency [1]. Decreased pancreatic hormones, including insulin, glucagon, and somatostatin, can deteriorate the control of serum glucose and ketone bodies [2]. Starvation ketosis is generally caused by fasting, eating disorders, or pregnancy [3]. It usually presents with mild hyperglycemia and rarely causes severe acidosis [4]. However, little is known about the presentation of starvation ketosis in patients with decreased pancreatic function.

## Case presentation

A 44-year-old Japanese man was admitted to the emergency department because of acute diarrhea, nausea, and mild abdominal pain. These symptoms were preceded by two days of inadequate oral intake due to adjustment disorder after changing his job. He had a medical history of alcoholic pancreatitis and pancreatic cyst for which he had undergone distal pancreatectomy two years earlier. His medication included pancrelipase, lansoprazole, and tramadol. Until two years earlier, he had been drinking 70 g of alcohol per day, but he denied any recent alcohol intake and illicit drug use. For confirmation, we heard from his wife about his behavior, who also denied alcohol and illicit drug consumption. On admission, his vital signs showed an axillary body temperature of 38.2℃, a heart rate of 124 beats per minute, a blood pressure of 137/80 mmHg, oxygen saturation of 99% on room air, and a respiratory rate of 27 breaths per minute. He looked unwell and was worried about his epigastric pain; however, physical examination revealed normal peristaltic sounds and no abdominal tenderness and was otherwise unremarkable. Arterial blood gas tests on room air revealed a pH of 7.33, a PCO_2_ of 29.7 mmHg, bicarbonate of 15.3 mEq/L, an anion gap of 27.5 mEq/L, lactate of 8.4 mmol/L, and glucose of 45 mg/dL (Table 1). Other blood tests revealed aspartate aminotransferase of 129 U/L, alanine aminotransferase of 49 U/L, amylase of 73 U/L, lipase of 12 U/L, and hemoglobin A1c of 7.6% (Table 2). The normal amylase and lipase levels were not suggestive of active pancreatitis. We did not measure his serum ketone levels. Dipstick urinalysis was positive for ketone bodies (Table 3). We did not measure his serum ethanol concentration because he and his wife separately denied his recent alcohol consumption. The urine drug test was also waived based on his current medical history.

**Table 1:** Results of the blood gas tests. Blood samples were obtained from the artery on Day 1 and from the vein on Day 3.

	Reference range	On admission (Day 1, arterial blood)	Two hours later (Day 1, arterial blood)	Day 3 (venous blood)
pH	7.35-7.45	7.33	7.35	7.35
PCO_2_ (mmHg)	35-48	29.7	22.1	47.6
PO_2_ (mmHg)	83-108	103	107	35.1
HCO_3_- (mmol/L)	22.2-28.3	15.3	12.1	25.7
Glucose (mmol/L)	3.6-5.3	2.5	7.6	16.3
Lactate (mmol/L)	0.56-1.39	8.4	11.4	3.6
Anion gap (mmol/L)	8-16	27.5	26.0	10.6
Base excess (mmol/L)	-3.2-1.8	-9.4	-12.3	0.8
Plasma osmolality (mOsm/L)	276-292	288	286	289

**Table 2:** Laboratory data of the blood samples.

	Reference range	Day 1	Day 2	Day 3	Day 6	Day 10	Day 13
White blood cell (/μL)	4,000-9,000	14,700	9,800	7,400	6,200	3,700	6,200
Hemoglobin (g/dL)	14.0-18.0	13.0	11.8	14.1	12.6	12.8	11.1
Platelet (×10⁴ /μL)	15.0-35.0	32.8	29.2	28.5	23.5	24.3	27.3
Total protein (g/dL)	6.7-8.3	7.6				6.9	
Albumin (g/dL)	3.8-5.3	4.6	4.4		4.9	4.2	4.1
Aspartate aminotransferase (U/L)	8-38	129	95	119	124	110	50
Alanine aminotransferase (U/L)	4-44	49	44	60	92	97	64
Lactate dehydrogenase (U/L)	124-222	235	216	258	197	412	191
γ-glutamyl transferase (U/L)	12-63				109	92	73
Sodium (mEq/L)	135-147	140	133	135	139	137	141
Chlorine (mg/dL)	98-108	93	94	95	98	100	100
Potassium (mEq/L)	3.6-5.0	3.1	3.9	3.9	3.7	4.6	4.2
Urea nitrogen (mg/dL)	8-20	11.4	8.7	8.2	8.7	4.6	4.2
Creatinine (mg/dL)	0.61-1.04	0.64	0.57	0.60	0.63	0.59	0.73
Amylase (U/L)	40-126	73					
Lipase (U/L)	13-42	12					
Hemoglobin A1c (%)	4.6-6.2	7.6					
C-reactive protein (mg/dL)	0-0.2	0.03	0.28	0.29	0.14	<0.03	<0.03
Prothrombin time (%)	80-120	119	124				

**Table 3:** Results of the dipstick urinalysis.

	Reference range	Day 1	Day 2	Day 3
Urine specific gravity	1.008-1.034	1.021	1.017	1.018
pH	4.8-7.5	5.5	6.5	6.5
Urine protein	-	±	±	-
Urine sugar	-	2+	-	4+
Urine ketone body	-	3+	1+	-

**Figure 1:**
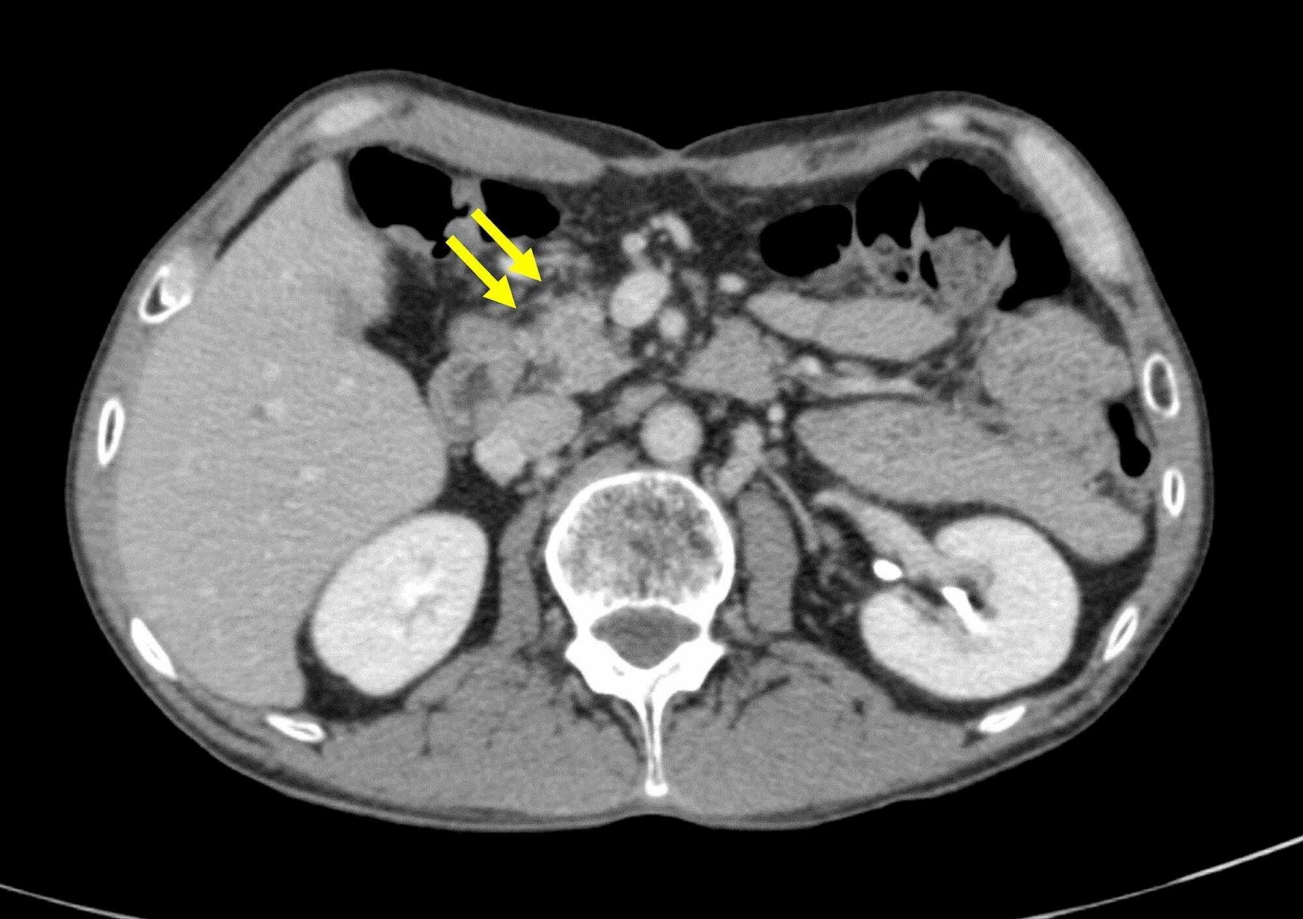
Computed tomographic scans of the abdomen with contrast.

Two sets of blood cultures on admission were negative. Computed tomography scans of the abdomen with contrast revealed no remarkable changes, including signs suggestive of active inflammation of the remaining pancreas (Figure 1). Intravenous glucose and vitamin B1 supplementation was administered in the emergency department, and nutritional management for presumed starvation ketoacidosis was begun. His symptoms improved gradually, but his premeal blood glucose level hovered at more than 200 mg/dL. A diagnosis of pancreatic diabetes was suspected based on decreased insulin secretion, normal insulin resistance, and negative anti-glutamic acid decarboxylase antibody (Table 4). Intensive insulin therapy was initiated, and he was discharged 15 days after admission. At his most recent visit, 30 days after discharge, he was asymptomatic and doing well.

**Table 4:** Laboratory tests to determine the type of diabetes mellitus. GAD, glutamic acid decarboxylase

	Reference range	Day 7	Day 10
Anti-GAD antibody (U/L)	0-5	<5	
Preprandial serum C-peptide (ng/mL)	0.69-2.45	0.85	0.51
Postprandial two-hour serum C-peptide (ng/mL)	-	2.74	
Preprandial serum insulin (μU/mL)	1.1-17.0	1.3	2.1
Postprandial serum insulin (μU/mL)	-	8.7	
Preprandial blood glucose (mg/dL)	60-109	152	123
Postprandial blood glucose (mg/dL)	-	362	

## Discussion

**Figure 2:**
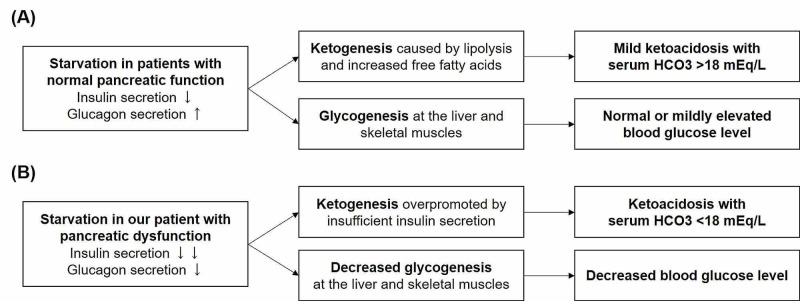
Pathophysiology of starvation ketosis. (A) In patients with normal pancreatic function, maintained insulin secretion usually prevents excessive ketogenesis, which results in relatively mild acidosis with the serum bicarbonate concentration not lower than 18 mEq/L. (B) In our case, the combination of chronic pancreatitis and starvation seemed to result in the strong suppression of insulin secretion and subsequent excessive ketogenesis.

Here, we described a case of hypoglycemia and severe ketoacidosis in a patient with chronic pancreatitis. As his current history was not suggestive of alcoholic or drug-induced ketoacidosis, his ketoacidosis was thought to result from two days of fasting, and serum ethanol measurement and urine drug test were waived. Starvation ketosis usually presents with normal or mildly elevated blood glucose levels because of decreased renal glucose clearance and enhanced counterregulatory hormones such as adrenalin, cortisol, and growth hormones (Figure 2A) [5]; however, our patient presented with hypoglycemia. Hypoglycemia is usually caused by the inappropriate use of hypoglycemic drugs and insulin therapy in patients with diabetes. Other differential diagnoses include cortisol deficiency, growth hormone deficiency, insulinoma, sepsis, hepatic failure, renal failure, liver failure, and chronic alcohol use [6]; however, these conditions were not present in our case. Idiopathic ketonic hypoglycemia is known to cause hypoglycemia and ketonuria in children who are non-diabetic and typically occurs in those under five years of age [7]; however, this disorder rarely occurs in adults. The combination of hypoglycemia and ketonuria in our patient may be due to decreased alpha cell function resulting from chronic pancreatitis (Figure 2B). Although the proportion of alpha cells in the islets is only about 20-30% [8], decreased alpha cells and glucagon secretion can result in a deteriorated defense mechanism against hypoglycemia. Additionally, decreased insulin secretion can cause a lack of glycogen storage in the liver and skeletal muscles [9]. This mechanism may also have contributed to hypoglycemia in our patient.

Our case is also notable for metabolic ketoacidosis with significantly decreased serum bicarbonate, which is uncommon among patients with starvation ketosis. In patients with starvation ketosis, the degree of acidosis is relatively mild, with the serum bicarbonate concentration not lower than 18 mEq/L (Figure 2A) [4]. This may be because insulin secretion in such patients is usually sufficiently maintained so as not to promote excessive ketogenesis [10]. In our patient, the combination of chronic pancreatitis and starvation seemed to have resulted in strong insulin secretion suppression and subsequent excessive ketogenesis, as seen in diabetic ketoacidosis (DKA) (Figure 2B). Recently, the concept of euglycemic DKA with a blood glucose level of less than 200 mg/dL has been proposed [4], with which the characteristics of our case seem to overlap.

## Conclusions

Short-term fasting can cause hypoglycemia in patients with decreased pancreatic function. It can also cause metabolic acidosis with significantly decreased serum bicarbonate, leading to severe illness and hospitalization. Starvation ketosis in our patient seemed to resemble DKA; thus, further reports should be accumulated to determine whether starvation ketosis accompanied by pancreatic dysfunction and DKA form an overlapping syndrome.
